# A Case of Torsion in an Otherwise-Normal Ovary with a Giant Hematosalpinx Larger than Enlarged Ovary: Utilization of Diagnostic Laparoscopy for the Accurate Diagnosis

**DOI:** 10.1155/2021/1371611

**Published:** 2021-08-23

**Authors:** Sae Yu, Koji Yamanoi, Masumi Sunada, Sachiko Kitamura, Naoki Horikawa, Yoshitsugu Chigusa, Ken Yamaguchi, Akihito Horie, Junzo Hamanishi, Eiji Kondoh, Masaki Mandai

**Affiliations:** Department of Gynecology and Obstetrics, Graduate School of Medicine, Kyoto University, 54 Shogoinkawahara-cho, Sakyo-ku, Kyoto City, Kyoto, Japan 606-8507

## Abstract

We report a case of torsion in an otherwise-normal ovary with a giant hematosalpinx. A 23-year-old woman presented with complaints of abdominal pain and nausea. At initial visit, there was few abnormal findings of imaging tests, and we made a diagnosis of ovarian hemorrhage. Three days later, she came back with increased symptoms, and we detected the mass of a complex solid cystic structure with a unilocular cyst much larger than solid component. A diagnostic laparoscopy was performed immediately, and we could make a diagnosis of torsion in an otherwise-normal ovary with a giant hematosalpinx. We performed a salpingectomy and could preserve her ovary. This is the first case of torsion in an otherwise-normal ovary with a giant hematosalpinx which enlarged to a greater extent than the ovary.

## 1. Introduction

The adnexal torsion is one of the most common gynecological emergencies. The ovary without an apparent tumor can even undergo torsion, known as a torsion in otherwise-normal ovary [1]. Because of the low frequency and similarity of symptoms to more common disease including ovarian hemorrhage, it is often difficult to accurately diagnose, and the diagnosis is reported to be often delayed [2, 3].

It is common in clinical practice to perform imaging tests to help to make an accurate diagnosis preoperatively. Since the adnexa receives blood flow from both the uterus and the gonadal arteries, the adnexal torsion would not completely block the blood flow into ovaries, and partial blockage of blood flow causes the blood stagnation, frequently resulting in the enlargement of the ovaries [4]. Namely, when there is an enlarged ovary on imaging examination, torsion of the ovary is considered as a differential diagnosis [4, 5]. In fact, it is reported that there are some abnormal findings in imaging tests including enlargement of the ovaries even in the otherwise-normal ovary torsion, along with decreased blood flow and changes in the position [6]. However, the imaging findings of fallopian tubes that are involved in the otherwise-normal ovary torsion are still unclear. In some cases, the fallopian tubes have been reported to become swollen and edematous due to involvement of torsion [7, 8], but to date, there have been no reports showing that the fallopian tube got larger than the ovary itself in case of torsion of otherwise-normal ovary.

Herein, we report a case of torsion in an otherwise-normal ovary, occurring in a nonpregnant woman of reproductive age. Left adnexal enlargement to approximately 10 cm, with an enlarged ovary and an even larger, cystic fallopian tube was documented. We performed the literature search on PubMed, using the keywords “adnexal torsion,” “hematosalpinx,” and “reproductive age.” However, we could not find any report of a markedly enlarged fallopian tube due to torsion in an otherwise-normal ovary. Because of these atypical imaging findings, we could not assume what the marked enlarged cystic tumor was derived from, and we could not understand the pathogenesis of her symptoms from imaging findings. A direct visual assessment with laparoscopy was performed, which enabled accurate diagnosis and prompt surgical treatment.

## 2. Case Report

A 23-year-old woman (gravida 0, para 0) presented to our emergency department with complaints of acute lower abdominal pain after sexual intercourse followed by nausea. She did not use any medication of contraception.

**Figure 1:**
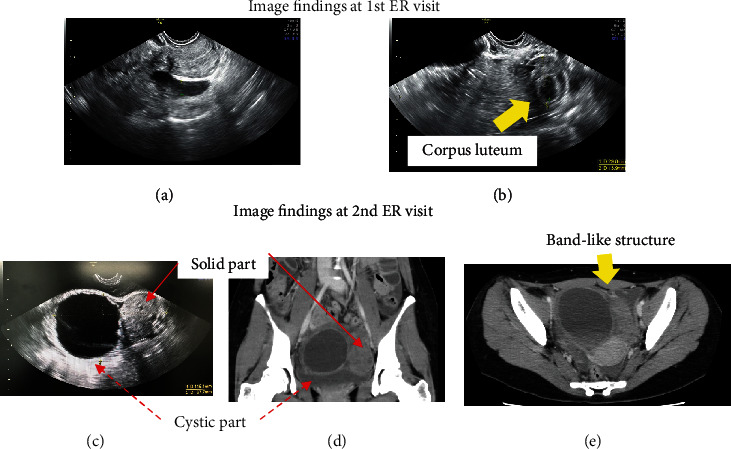
Image finding at 1st ER visit and 2nd ER visit. (a, b) Image findings at 1st ER visit. (a) Small amount of ascites on the left side. (b) A left adnexa 3 cm in size, and a small cyst inside that was suspected to be a corpus luteum. (c–e) Image findings at 2nd ER visit. (c, d) There are two-follicular enlargement. (e) A band-like structure between the solid and cystic parts. Arrows are indicated.

Physical examination revealed a soft and flat abdomen with no tenderness in the pouch of Douglas. Blood examination showed slightly elevated white blood cell (WBC) count 9550/*μ*l, C-reactive protein (CRP) 2.3 mg/dl, and a negative human chorionic gonadotropin. On ultrasound, left adnexa was not enlarged, and a corpus luteum-like cyst, 3 cm in size, was detected inside the ovary. Small amount of ascites was also observed (Figures 1(a) and 1(b)).

Based on the above findings, provisional diagnosis of ovarian hemorrhage was made, and conservative treatment with oral painkillers was administered.

Three days later, the patient returned to the emergency department with increased intensity of lower abdominal pain along with strong nausea and diarrhea. Her vitals showed an increased pulse rate of 103 bpm and body temperature of 37.7°C. Her abdomen was generally soft, but there was moderate tenderness in the pouch of Douglas. Blood examination showed elevated inflammation-related factors (WBC 11,510/*μ*l, CRP 12.0 mg/dl). Her platelet counts were kept in normal range (21.6 × 104/*μ*l at her 1st visit and 25.6 × 104/*μ*l at her 2nd visit). Ultrasonography revealed a markedly enlarged adnexal mass (>10 cm × 15 cm) in the left lower abdomen. The mass had a complex solid cystic structure with a unilocular cyst much larger than solid component (Figure 1(c)). Contrast-enhanced computed tomography (CT) was then performed. The mass, located on the ventral side of the uterus, showed a weak contrast effect and a separate cystic compartment connected to the solid area with a band-like structure (Figures 1(d) and 1(e)).

Based on the above findings, adnexal torsion was suspected; however, the composition of enlarging cystic mass and the reason of rapid enlargement over a few days were unclear although we discussed it fully with radiologists. Nonetheless, to rule out ovarian torsion, a laparoscopic surgery was performed immediately.

**Figure 2:**
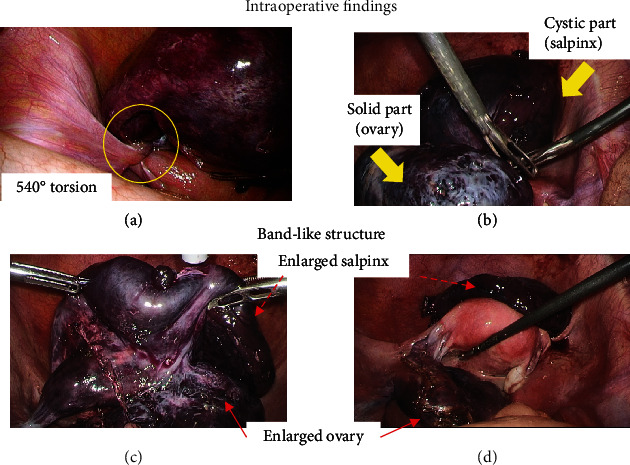
Intraoperative findings. (a) Adnexal torsion was observed. (b) Two-follicular enlargement was observed. (c) A band-like structure was observed. (d) Left salpingectomy was performed. Arrows are as indicated.

On laparoscopic surgery, the whole left adnexa was found to be markedly swollen and twisted to at least 540 degrees (Figure 2(a)). Detorsion of the adnexa was performed, and separate cystic and solid masses were visualized. On aspirating fluid from the cystic mass, fimbria-like structure was observed on the surface of cystic mass (Figure 2(b)) suggesting that the cystic mass was a hematosalpinx and the solid mass, an enlarged ovary. A band-like tissue was connected the solid ovary to the fimbria (Figure 2(c)). A left salpingectomy was performed because of ischemic, irreversible changes in the enlarged left fallopian tube. Considering the young age of the patient and the weak contrast effect seen in CT, the ovary was preserved (Figure 2(d)). Total duration of the procedure was 1 h and 49 min, and total blood loss was 300 ml (including bloody ascites during laparoscopy).

**Figure 3:**
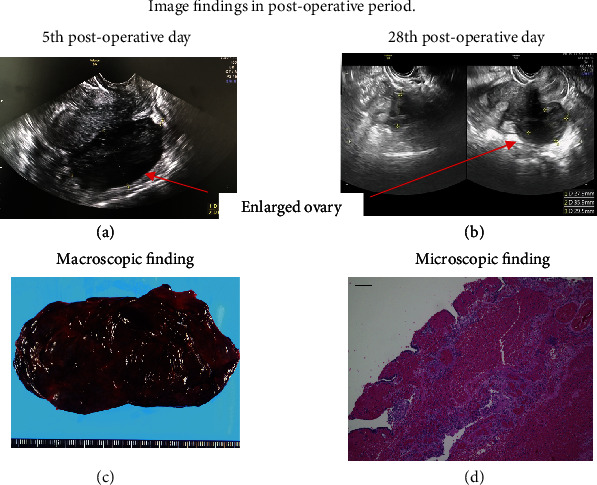
Image findings in postoperative period. (a, b) Ultrasonography findings. (a) An ultrasonography finding on 5th postoperative day. (b) An ultrasonography finding on 28th postoperative day. Arrows: left ovary. (c, d) Pathological findings. (c) Macroscopically, cyst was filled with blood. (d) Microscopically (40x), extensive necrosis and congestion of the cyst wall with some preserved fallopian tube epithelium were observed. Scale bar: 100 *μ*m.

After surgery, her symptoms (abdominal pain and nausea) disappeared quickly. Laboratory tests showed a rapid decrease in inflammatory markers. On the fifth postoperative day, the left ovary measured 62 × 36 mm (Figure 3(a)), and the patient was discharged on the same day. On 28th postoperative day, physical examination was unremarkable, and the left ovary had shrunk to 35 × 29 mm in size (Figure 3(b)).

Grossly, the cyst was unilocular, filled with blood, and had no obvious mass lesion (Figure 3(c)). Microscopically, extensive necrosis and congestion of the cyst wall with some preserved fallopian tube epithelium were observed (Figure 3d). Hence, a definitive diagnosis of fallopian tube hematoma due to adnexal torsion was made.

## 3. Discussion

We encountered a case of torsion in an otherwise-normal ovary in a nonpregnant woman of reproductive age with a marked enlarged hematosalpinx.

Especially in a nonpregnant woman of reproductive age, it is difficult to distinguish adnexal torsion from other common conditions with similar symptoms [9]. In this case, we first suspected ovarian hemorrhage because the patient was on cycle date 12 at the time of first visit and stated that she had sexual intercourse before development of symptoms. However, large ascites (intraperitoneal hemorrhage), which is often seen in ovarian hemorrhage [10], was not observed at this time. When a sudden abdominal pain occurs in a young woman and there remains some unclear findings, otherwise-normal ovarian torsion should be included in the differential diagnosis, and careful follow-up is recommended.

Because her left adnexa was not swollen at her first visit, her left fallopian tube as well as the ovary is thought to get very enlarged due to torsion. In a case report by Kamio et al., enlarged fallopian tube was seen as a separate structure from the ovary, but the size of the fallopian tube was smaller than that of the ovary [11]. In our case, on the contrary, the cystic fallopian tube formed an adnexal mass along with the ovary and was found to be enlarged to a greater extent than the ovary. Since the adnexa receive dual blood supply from the uterine and gonadal arteries, adnexal torsion can lead to partial block resulting in blood stagnation and enlargement of the ovaries [5, 12]. We propose that tube enlargement was the result of blood flow obstruction caused by adnexal torsion. Although the reason behind this marked fallopian tube hematoma formation due to congestion of blood flow still needs to be elucidated, it should be noted that the fallopian tubes can preferentially get enlarged when they are involved in the torsion of otherwise-normal ovary.

At first, the large cystic mass did not look like a fallopian tube, and it confused us a lot. In case of a torsion of otherwise-normal ovary, the imaging findings about fallopian tube can vary like our case. Clinically, it is very important to suspect a torsion of otherwise-normal ovary if we find the enlargement of ovary, decreased adnexal blood flow, and atypical position of the ovary [5, 13], regardless of other findings.

Whenever adnexal torsion is suspected, appropriate investigations are needed to rule out the condition. Delay in diagnosis and therapeutic intervention may lead to abolition of ovarian function. In case of adnexal torsion, we usually avoid oophorectomy to preserve ovarian function [14]. Especially in case of a relatively young age, we should try to preserve ovarian function to prevent loss of fertility [15].

Laparoscopy is a useful tool for the accurate evaluation of several intra-abdominal pathologies because it enables observation of the entire abdominal cavity with a relatively small wound [16, 17]. Also, emergency laparoscopy has the advantage of minimizing invasiveness and cost, as well as maximizing patient comfort in terms of pain control and postoperative recovery [18, 19]. However, as the field of view is limited, it can be difficult to observe the large structures fully, as in our case. In addition, if the tissue is highly fragile due to torsion, as in our case, rough handling may result in inadequate observation of the lesion due to contusion or bleeding from the tissue. Hence, it is important to carefully handle the tissues to maintain a clear field of vision. Additionally, in case of cystic structures, aspirating the cyst fluid may enhance visualization of the target tissue and associated unique structures, hence providing a diagnostic clue.

## 4. Conclusion

We encountered a case of torsion in an otherwise-normal ovary with a giant hematosalpinx. A marked cystically enlarged fallopian tube made it difficult to fully understand the actual pathophysiology. Because diagnosis of otherwise-normal ovary torsion is often difficult, we should not rule out its possibility. If we suspect it from any reason, we should consider treating it without delay to preserve their fertility. For treatment, laparoscopic surgery is useful because the lesion can be observed in detail with a small wound, and treatment can be performed sequentially.

## Data Availability

The data that support the findings of this study are available from the corresponding author upon reasonable request.
